# Asthma: Expanding the Medicine Chest

**DOI:** 10.1289/ehp.114-a96b

**Published:** 2006-02

**Authors:** Michael Szpir

About 10% of the asthmatic population has a severe form of the disease that can require progressively higher doses of corticosteroid drugs to manage the symptoms. Now a new therapeutic approach, described in the December 2005 issue of *Thorax*, may help people whose severe asthma symptoms no longer respond to steroid treatments.

Early research suggested that asthma was a so-called Th2 cytokine disorder involving certain white blood cells known as eosinophils. However, Stephen T. Holgate, a professor in the Infection, Inflammation, and Repair Division of Southampton General Hospital in the United Kingdom, and other researchers noticed that severe asthma was actually associated with neutrophils, another type of white blood cell that is associated with Th1 diseases such as rheumatoid arthritis and psoriasis. These diseases respond well to treatments that block the action of an immune system molecule called tumor necrosis factor–alpha (TNF-α). If severe asthma was truly a Th1 disease, Holgate hypothesized, it stood to reason that it too would respond to a TNF-α blocker.

To test this idea, Holgate and his colleagues administered the drug etanercept (Enbrel®) to 17 subjects with severe asthma in a 12-week study. Etanercept is a soluble receptor that binds to TNF-α. Treatment was associated with significant improvements in asthma symptoms and lung function, and reduction of bronchial hyper-responsiveness (abnormal sensitivity to agents that narrow the airways) in the 15 patients who completed the regimen.

Some research now suggests that asthma is not a single disease. “Mild and moderate forms of asthma may be disorders primarily characterized by a Th2-type immune response associated with allergen-specific IgE antibodies,” Holgate says. “In contrast, severe asthma, which is aggravated by viruses and air pollution, may be a separate Th1-type immune disorder that involves the excess production of TNF-α.” This could explain why the use of etanercept in previous studies of mild asthma produced no improvement in symptoms.

Although the improvement in asthma symptoms and airway hyperresponsiveness are impressive, placebo-controlled studies are needed to assess the efficacy of anti-TNF-α therapy. According to Holgate, such studies are now under way in his and other laboratories, and the preliminary results look quite promising. “In twenty-seven years of asthma research, this is the biggest breakthrough that [our research group has] had,” he says.

## Figures and Tables

**Figure:**
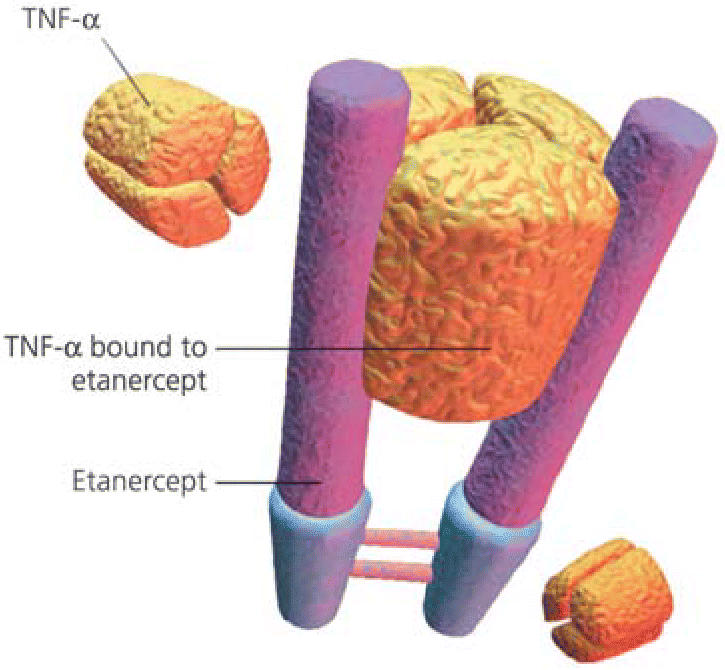
Asthma attacker? The drug etanercept binds to TNF-α to block its action on the immune system. The drug may be an effective treatment for severe asthma aggravated by factors that affect immunity, such as viruses and air pollution.

